# Integrated care reform in urban China: a qualitative study on design, supporting environment and implementation

**DOI:** 10.1186/s12939-017-0686-8

**Published:** 2017-10-25

**Authors:** Yi Qian, Zhiyuan Hou, Wei Wang, Donglan Zhang, Fei Yan

**Affiliations:** 1 Department of Social Medicine, School of Public Health, Fudan University, P.O.Box 250, 138 Yi Xue Yuan Road, Shanghai, 200032 China; 2 Key Lab of Health Technology Assessment, National Health and Family Planning Commission of the People’s Republic of China, Fudan University, Shanghai, 200032 China; 3 Collaborative Innovation Center of Social Risks Governance in Health, Fudan University, Shanghai, 200032 China; 4 Department of Health Policy and Management, College of Public Health, University of Georgia, Athens, GA 30602 USA

**Keywords:** Integrated care, Health care delivery system, Primary health care, Qualitative study, China

## Abstract

**Background:**

Initiatives on integrated care between hospitals and community health centers (CHCs) have been introduced to transform the current fragmented health care delivery system into an integrated system in China. Up to date no research has analyzed in-depth the experiences of these initiatives based on perspectives from various stakeholders. This study analyzed the integrated care pilot in Hangzhou City by investigating stakeholders’ perspectives on its design features and supporting environment, their acceptability of this pilot, and further identifying the enabling and constraining factors that may influence the implementation of the integrated care reform.

**Methods:**

The qualitative study was carried out based on in-depth interviews and focus group discussions with 50 key informants who were involved in the policy-making process and implementation. Relevant policy documents were also collected for analysis.

**Results:**

The pilot in Hangzhou was established as a CHC-led delivery system based on cooperation agreement between CHCs and hospitals to deliver primary and specialty care together for patients with chronic diseases. An innovative learning-from-practice mentorship system between specialists and general practitioners was also introduced to solve the poor capacity of general practitioners. The design of the pilot, its governance and organizational structure and human resources were enabling factors, which facilitated the integrated care reform. However, the main constraining factors were a lack of an integrated payment mechanism from health insurance and a lack of tailored information system to ensure its sustainability.

**Conclusions:**

The integrated care pilot in Hangzhou enabled CHCs to play as gate-keeper and care coordinator for the full continuum of services across the health care providers. The government put integrated care a priority, and constructed an efficient design, governance and organizational structure to enable its implementation. Health insurance should play a proactive role, and adopt a shared financial incentive system to support integrated care across providers in the future.

**Electronic supplementary material:**

The online version of this article (10.1186/s12939-017-0686-8) contains supplementary material, which is available to authorized users.

## Background

China launched the national health care reform in 2009, which aimed to provide affordable and equitable health care to all citizens [1]. Currently, China has achieved impressive progress in the health insurance system which covered more than 95% of the country’s population, but health care delivery system has become a fragmented and hospital-centered framework rather than an integrated one that focuses on primacy health care (PHC) [1–4]. Although more health resources have been allocated to strengthen PHC during the health care reform, patients still preferred hospitals instead of PHC providers. It is common in China for patients to be on long waiting lists at hospitals, while at the same time, fewer patients visited PHC facilities. From 2009 to 2014, the proportion of outpatient services provided by hospitals among all outpatient services continued to increase from 30.8% to 35.9%, and the proportion of admissions to hospitals even remarkably grew by 11.5% (from 61.7% to 73.1%), implying that PHC providers did not play the role of gatekeepers for health care [5–10]. As a result, the considerable resources invested by the government in health care reform have been disproportionately flowed into hospitals instead of PHC facilities [1, 2, 4]. Nudging and incentivizing patients to seek health care at PHC facilities continued to be a priority in the new round of China’s health care reform [11].

Meanwhile, China has been experiencing dramatically demographic and epidemiological transitions, which challenged the current health care system. In 2014, there was around 9% of Chinese population aged 65 and over [12], and this proportion was predicted to reach 20% in 2030 [1]. Additionally, there were about 260 million patients with noncommunicable diseases (NCD) in 2012, accounting for 87% of annual deaths [13, 14]. It was estimated that the accumulated economy loss of lives due to stroke, heart diseases, and diabetes could reach $556 billion in China from 2005 to 2015 [15]. Apparently, the current hospital-centered and fragmented delivery system is incapable to meet the health needs of the rapidly increased aging population, weakens continuity of care, and leads to cost escalation [11]. Therefore, a more effective and efficient health care model is necessary to improve health for NCD patients.

Internationally, integrated care was initially introduced to address the fragmentation in health services, and facilitate the coordinated and continuous care for NCD patients [16–22]. It has been increasingly adopted in many countries to provide more continuous and cost-effective health care to elderly populations or to subpopulations with chronic diseases [1, 2, 19]. Some evidence has reported the benefits of integrated care in improving quality of care [1, 23, 24], patient satisfaction [23], access to care [22, 25], and managing demand [26], but no consistent findings in its economic impact [23, 26, 27]. In our study, integrated care refers to the definition given by World Health Organization: "the management and delivery of health services so that clients receive a continuum of preventive and curative services, according to their needs over and across different levels of the health care system" [20]. In particular, we focused on the vertical coordination between hospitals and PHC facilities to deliver a broad package of preventive and curative health services with primary health care playing the key role for a target population [16–20].

Recently, China initiated some pilot models of integrated care between hospitals and community health centers (CHCs) [16, 28–30]. The integrated care delivery model in rural Henan Province, which developed a vertical referral system among health facilities through the case-based payment and an integrated information system for sharing patients’ information within the system, was found to have significant impact on changing healthcare seeking behavior and improving the accessibility, continuity and coordination of health care for NCD patients [11]. In Huangzhong County, Qinghai Province, the consortium consisting of one county hospital, several CHCs and many village clinics was developed for schizophrenia and diabetes patients, which showed no remarkable improvement on healthcare coordination [31]. The authors also identified a lack of motivation for care coordination, insufficient health professionals, and inadequate supports as the main barriers for integration of healthcare system in Huangzhong County [11]. As one of these pilots, the Hangzhou municipal government has implemented comprehensive interventions, including improvement of the capacity and quality of health services in CHCs, and the coordination between CHCs and hospitals [32–35]. An integrated care model, namely the Joint Health Center (JHC) for chronic care, was created in Hangzhou in this context. These pilots have achieved some progress but also faced several impediments during the process of implementation, which provided important experiences and lessons for further scaling up of integrated care models in China. However, there was limited evidence on these integrated care pilots and to date no research has been done to explore the implementation of these pilots and perspectives from various stakeholders (policy makers, administrative staff and medical staff participated in these pilots, and patients) [30, 36–40].

Our research questions were: (1) what perspectives the stakeholders held on the integrated care reform, and (2) what aspects or factors facilitated or constrained the integrated care reform. Taking the JHC model for chronic care from Hangzhou as a case example, this study adopted qualitative methods to investigate stakeholders’ perspectives on its design features and supporting environments, their acceptability of this pilot, and further identify the enabling and constraining factors that may influence the implementation of the integrated care reform. This study would be useful to identify and solve the constraining factors in the future implementation and scale up the integrated care reform in China and in other countries with similar context.

## Methods

### Study design and framework

**Fig. 1:**
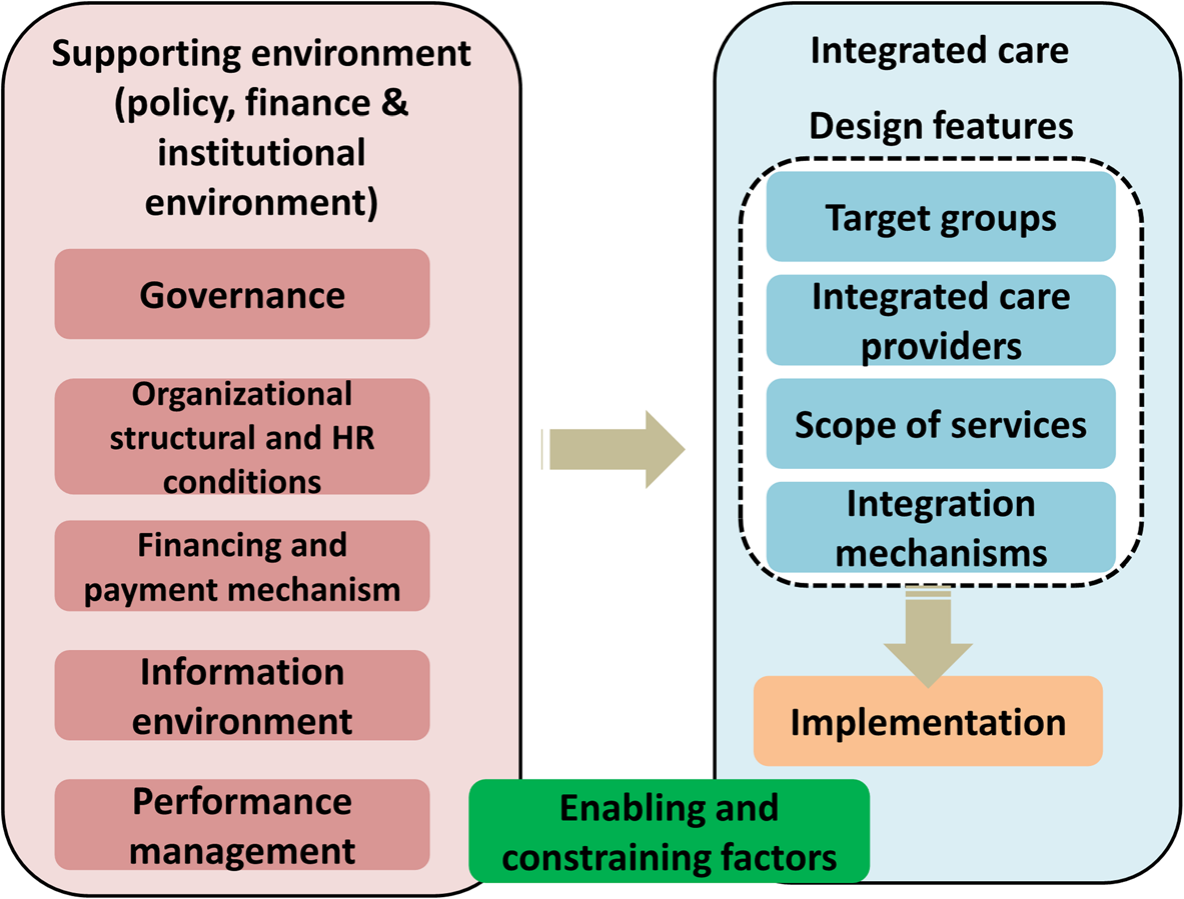
Analytic framework of the integrated care pilot in Hangzhou, China

Figure 1 showed our analytic framework for the integrated care reform [19, 41]. The design feature of an integrated care model can be analyzed from the following aspects: target groups, integrated care providers, scope of services, and integration mechanisms; and the supporting environment can be structured into five aspects: governance, organizational structure and human resources (HR) conditions, financing and payment mechanism, information environment, and performance management. Each aspect can play as enabling or constraining factors for the implementation of the integrated care model.

The JHC policy documents were collected and used to describe the design feature of JHC model. And we applied qualitative methods, including in-depth interviews (IDI) and focus group discussions (FGD) with key informants, to investigate the perspectives on the supporting environments and implementation of JHC from policy makers, administrative staff and medical staff from health facilities, and NCD patients. These perspectives were used to explore the influencing factors in its implementation, and identify which aspects abovementioned enable or constrain the integrated care reform.

### Study setting

Hangzhou, the capital city of Zhejiang Province, is located in the eastern and coastal area of China. There were 8.89 million residents in 2014, with 98.9% covered by public health insurance, and the yearly disposable income per capita was 5893 US dollars [42, 43]. The health care delivery system in Hangzhou is a typical three-tiered system, consisting of CHCs, secondary and tertiary hospitals. In 2013, there were 4139 health facilities totally [43, 44]. The average number of total medical beds and hospital beds per 1000 people were 7.37 and 6.60. And the average number of total health professionals, physicians (including physician assistants) and nurses per 1000 people were 11.09, 4.20, and 4.39 respectively [43, 44]. More information of health resources in Hangzhou were presented in Additional file 1: Table S1.

### Sampling of key informants

A total of 50 key informants (27 male, 23 female) were purposely recruited, among those who were involved in the policy-making process and implementation of the JHCs (Table 1). There were four types of key informants: policy makers, administrative staff and medical staff from health facilities in the JHC’s service network, and NCD patients. Policy makers were from Zhejiang provincial and Hangzhou municipal government agencies, including the Development and Reform Commission, the Health and Family Planning Commission, the Department of Finance, and the Department of Human Resource and Social Security. One hospital and two CHCs were selected specifically due to their rich experiences in participating in the JHCs. The characteristics of the sampled key informants were presented in Additional file 1: Table S2a and S2b.

**Table 1:** Number of sampled key informants

Organizations	Policy makers	Administrative staff	Medical staff	Patients with chronic diseases
Provincial government	4	–	–	–
Municipal government	9	–	–	–
Hospitals	–	7	6	–
Community health centers	–	8	8	8
Total	13	15	14	8

### Data collection

The fieldwork was carried out at Hangzhou in January, 2015. The policy documents on integrated care reform were collected from Zhejiang province and Hangzhou city. In addition, topic guides for different types of key informants were applied to facilitate the IDI and FGD. The topic guides were designed based on the analytic framework, including design feature (further grouped into four aspects), supporting environment (further grouped into five aspects), and implementation (Additional file 1).

The FGD or IDI was used according to the type of key informants. FGD aimed to inspire more discussion among the same type of key informants, which only contained one type of key informant and was used for policy makers, administrative staff and medical staff respectively. IDI aimed to get perceptions in a greater detail or the privacy issue, which was used for key policy makers, the director of health facilities, chief general practitioners (GP), and patients. For the policy makers, we started with FGD including all the policy makers (Development and Reform Commission, Health and Family Planning Commission, Department of Finance, and Department of Human Resource and Social Security) to investigate the design feature and supporting environments since the implementation of JHC was a collective decision. And then we moved on to do IDI on key policy makers (Health and Family Planning Commission who is in charge of health care, Department of Human Resource and Social Security who is in charge of health human resources and health insurance) to get more in-depth details on the topics that appeared in FGD and also their acceptability. For the health care facilities, we also started with FGD for administrative and medical staff, and then moved on to do IDI on key administrative and medical staff (director and chief GP). Directors of health facilities were arranged for IDI because they had the higher hierarchy than other administrative staff, who could potentially influence the responses of other administrative staff if they were in the same FGD. The chief GPs were arranged for IDI because they were responsible for the integrated care. For NCD patients, only IDI was conducted due to the privacy issue.

The research team had attended the training on qualitative research and had experiences with field work for qualitative studies. The IDI and FGD were conducted in Mandarin, and most of them lasted for 2 h and were terminated when there was no new information forthcoming. During the FGD, the facilitator tried to make sure the perspectives from each participant were well-captured. The final sample size was reached till saturation of information. There were 9 FGDs and 13 IDIs facilitated separately. All of them were audio recorded and the field notes were taken.

### Data analysis

The recordings were verbatim in Mandarin to prevent loss of meaning, and then counterchecked by a research assistant for accuracy of the transcriptions. A thematic analysis was employed to analyze the qualitative materials using the MAXQDA 10 software (Cologne, Germany). The analysis process was conducted as following: going through all the text data and materials collected for familiarisation; noting down and refining the recurring issues and concepts; developing the initial themes and codes; assigning the texts to relevant themes and codes. The themes were generated from the topic guides, the textual data from IDI and FDG, field notes, and policy documents. The themes and codes were revised and refined continuously with integration of new insights. Two researchers conducted the analysis independently, and then compared the results, and discussed the differences until an agreement was achieved. The typical quotes and their identifiers were provided in the results section below, which were translated into English and also checked by a coauthor for accuracy.

## Results

### Design features described in policy documents

The JHC for chronic care was initiated by the Hangzhou municipal government as a pilot project of integrated care in 2013. It was introduced to improve the capacity and quality of chronic diseases management in primary care settings. The design features of this pilot were defined based on the policy documents (Table 2).

**Table 2:** Design features of Joint Health Center (JHC) for chronic care in Hangzhou City

Key aspects	Description
Target population	NCDs patients, with a particular focus on diabetes and hypertension patients at the initial stage.
Provider network	Four tertiary hospitals and forty-six CHCs participated the providers network. The networks were constructed according to the geographic location, and each tertiary hospital was designated to collaborate with the CHCs in the same or nearby district.
Organizational structure	Each CHC was responsible for the establishment of a local JHC based on its existing medical resources, especially for the preparation of a well-equipped consultation room for integrated care. Forty-six JHCs with a unified logo were founded in the CHCs.
Healthcare personnel	The CHC’s director was assigned to be the JHC’ director, in charge of its operation. Directors of hospital were in charge of the coordination with CHCs and selection of specialists. The JHC had a team of medical staffs consisting of the chief general practitioners (GPs) and nurses from CHCs, and specialists from hospitals. Chief GPs from CHCs played the gatekeeper role and guided NCDs management, and specialists from hospitals collaborated with chief GPs to provide integrated care and to train chief GPs. An innovative mentorship system was first introduced between chief GPs and specialists.
Integration mechanism	The CHCs and hospitals signed the cooperation agreement for NCDs management. The primary care and specialist care were integrated and delivered to the NCDs patients through the JHCs.
Scope of services	The scope of services included the integrated care of primary health care and specialist care in the JHCs, and the care in coordinated hospitals. The patients were reasonably referred to different levels of care facilities based on their medical conditions and were followed up by their chief GPs.

Notes: CHCs: community health centers; NCDs: non-communicable diseases; GPs: general practitioners

According to geographic location, four tertiary hospitals and forty-six CHCs (about 36% of total CHCs) in Hangzhou participated in this pilot to establish the integrated care providers network. Each CHC was responsible for the establishment of a local JHC based on its existing medical resources. The JHC had a team of medical staffs consisting of the chief GPs and nurses from CHCs, and specialists from hospitals. Note that an innovative mentorship system was first introduced between chief GPs and specialists. In the mentorship system, each specialist was designated to be the supervisor of several chief GPs and coached and supported them for NCD management. Since one hospital collaborated with many CHCs, one specialist usually needed to work with several chief GPs.

**Fig. 2:**
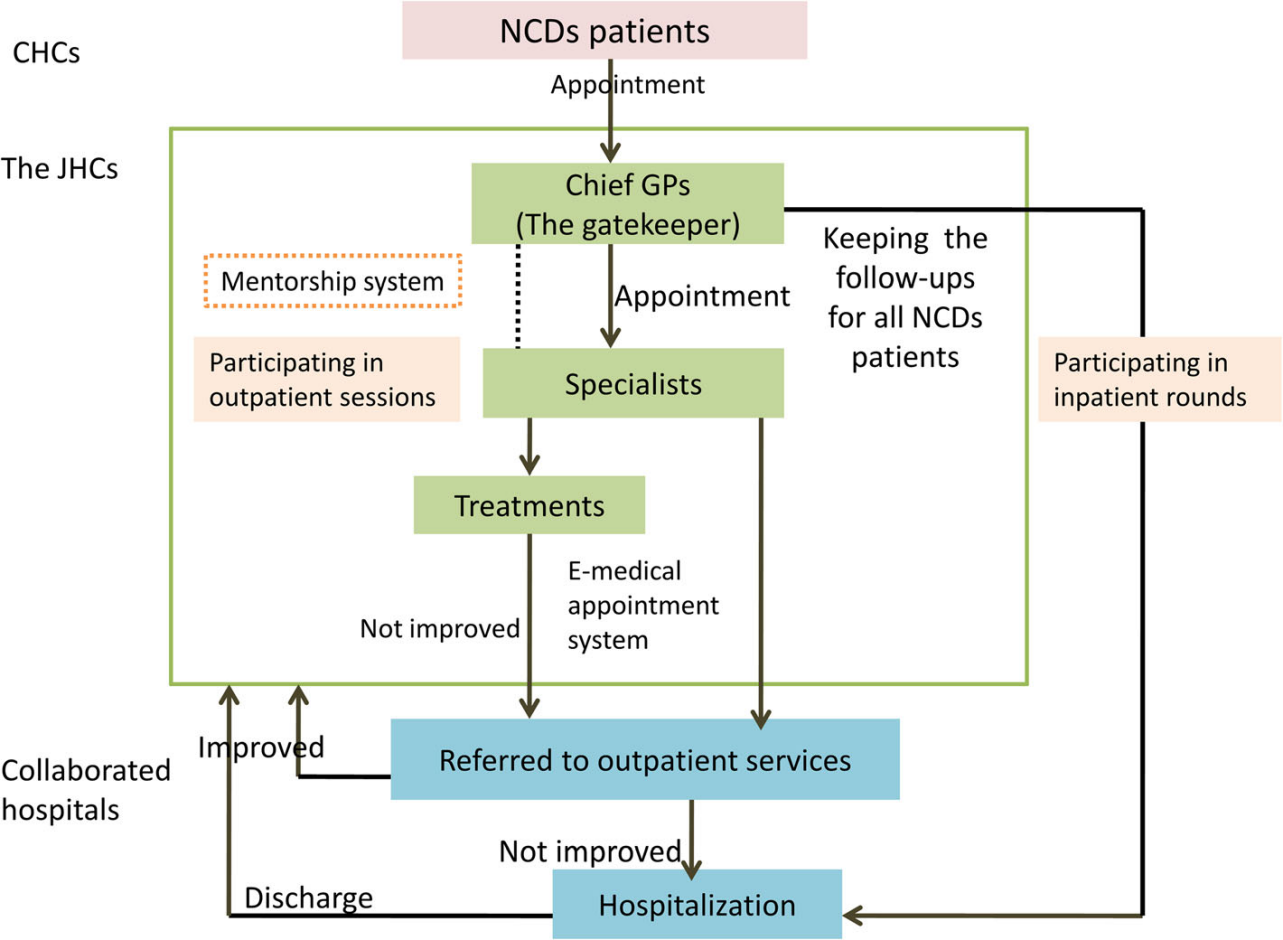
The integration mechanism and care delivery in the Joint Health Centers (JHCs). Notes: CHCs: community health centers; NCDs: non-communicable diseases; GPs: general practitioners

The primary care and specialist care were integrated and delivered to the NCD patients through the JHCs (Fig. 2). The chief GPs firstly screened the NCD patients who may need further treatments, and assisted the patients to make appointments with the specialists. The specialists then visited the JHCs to provide the consulting services, while chief GPs also participated in the outpatient sessions for follow-up services and learning. For those who needed further examinations or treatments, they would be referred to the collaborated hospitals through an E-medical appointment system and be referred back to the JHCs if their conditions got improved. The chief GPs were required to attend the inpatient sessions for the referred patients and also provided follow-up services. The GPs and nurses assisted the chief GPs and specialists in this process.

However, there were some uncertainties influencing the implementation of JHC. Firstly, there was a lack of clarification on the integrated care standards that the chief GPs and the specialists could follow for NCD patient management, such as clinical protocols and disease management programs, and it was unclear how the CHCs and hospitals could support the referrals. Moreover, the policy did not define the funding mechanism of the JHCs. There was also a lack of specific requirements for medical equipment needed in each JHC, and the only statement about that in the policy documents was "to have basic medical equipment in each JHC".

### Perspectives on the JHCs’ supporting environment from the key informants

#### Governance

##### Building the leadership team using a top-down structure

All policymakers stated that the government highly valued this integrated care model and strengthened the leadership. The leadership team was established, and it consisted of the directors of municipal and district health departments, the specialist team, and collaborative directors from CHCs and hospitals. The policymakers highlighted that the leadership team had developed an effective top-down governance structure, which was considered as one key enabling factor in implementation.

##### Adequate autonomy for CHCs

The majority of the administrative staff of healthcare providers mentioned that the CHCs had their own decision rights on the development and operation of the JHCs, which enabled them to play the core role in the integrated care model. Many interviewees from the CHCs thought that adequate autonomy significantly motivated their participation in the initiatives.

### Organizational structure and HR conditions

#### Innovation of organizational structure

Both administrative and medical staff agreed that as an organizational innovation, the JHCs were established based on CHCs using the current medical resources, which created a new linkage that integrated the primary care and specialist care in a cost-effective way and strengthened the gatekeeper role of the chief GPs. The CHCs took the main responsibilities for creating the JHCs, and the hospitals provided the support and supervisory. However, the administrative staff of healthcare providers stated the integration of this pilot only occurred on healthcare provision at the JHCs, but not on the administrative integration across providers, implying the hospitals and CHCs had independent authorities on finance and administration. Organizing meetings and discussions were reported as the main communication approaches between hospitals and CHCs. And some administrative staffs were reluctant about the JHCs because they thought the current integration was only based on the cooperation agreement bonded between physicians rather than on official contracts between providers.

#### Selecting the qualified staff from the existing pool of health personnel

According to the policymakers and administrative staff, this pilot recruited qualified staff from the existing pool of health personnel based on certain criteria rather than employing new staff. The leadership teams, the chief GPs, the specialists and other staff were all current employees of the health care system. However, this pilot did not have any autonomy over the hiring and firing decisions on these staff who worked only part-time in this integrated care pilot and spent most time doing their original work. Some medical staff in tertiary hospitals considered that their workload increased much after participation in the JHC initiative.

*"The government requires us to select the qualified staff for this project, so we select the ones who meet the requirements. Not all the specialists of cardiovascular diseases and endocrine diseases in our hospital are eligible, and we usually select the best specialists who are also good at teaching. They need to go to the JHCs regularly. We would also respect the individual willingness to join this project, but some specialists are too busy to participate in the project." (From one administrative staff in a hospital).*

*"As a senior physician in the hospital, I need to provide outpatient and inpatient services, and are also required to be on-call every 6 days. Now I also participate in the JHC, so sometimes, I feel too tired..." (From one medical staff in a hospital).*

### Innovative capacity building mechanism - the mentorship system

Most informants had positive perspectives on the mentorship system and considered it one main facilitator for implementation. The mentorship system was perceived to be better than the previous capacity building programs because it built a one-on-one relationship between the chief GP and specialist, which enabled the convenient and effective communication between the trainee and trainer. The chief GPs considered it as a learning-from-practice model to proactively participate in the training, through which they could directly consulted their mentors and also attended the outpatient and inpatients sessions.

*"Our chief GPs could go to hospital for attending the inpatient rounds, and they [specialists] would come to our JHC to provide outpatient care consultation as well. We really like this new training mechanism. It is better than the previous trainings. We have mentors, and we can ask questions and get supports from them at any time. Previously we were simply sitting at the training classes..." (From one chief GP).*

### Financing and payment mechanism

#### Lack of shared financial incentives and payments across providers

The administrative staff stated that there was no specific financing and payment mechanism developed for this pilot. Health care providers still had independent budgets and there was no resource pooling to allow budget flexibility across providers. Furthermore, there was also lack of shared financial incentives across providers to encourage the integration of care, and this may discourage them from working together.

#### Insufficient financial incentives provided to the medical staff

The policymakers mentioned that there was a lack of explicit policies that provided rewards to medical staff for participating in this pilot. The specialists could receive few subsidies from the hospitals and CHCs for outpatient services at the JHCs, but the subsidies from CHCs were not on a regular basis, depending on the financial situation of the CHCs. Moreover, the policymakers also said that health insurance did not cover the specialists’ consultation fee when working in JHCs. This largely discouraged some specialists to attend the outpatient sessions in JHCs. Additionally, the chief GPs mentioned that they did not receive any financial rewards directly but their work in the JHCs would be taken into consideration for their performance evaluations. Also, there was no financial incentive for patient referrals.

*"We do not arrange the financial rewards for the specialists into the policy. The health care providers could decide how much they would reward to specialists..." (From a policymaker).*

*"We usually provide certain subsidies to specialists for their outpatient services. We think we should pay for their contributions, but some CHCs don't provide any subsidies, since there are difference financial situations..." (From administrative staff of CHC).*

### Little influence of health insurance on patients’ health care seeking behavior

Many medical staff mentioned that in the current health insurance system, patients had free choices to seek care at any level of health care facilities. Health insurance had little restrictions on patients’ health care seeking behaviors, and patients still could bypass the CHCs and JHCs to access care in hospitals. According to the Urban Employee Basic Medical Insurance (UEBMI), the reimbursement rates for outpatient services in CHC and tertiary hospitals were close (88% and 76% respectively, with the same deductibles). Even under the Urban Residents Basic Medical Insurance (URBMI), patients could receive much more reimbursement for outpatient services in CHC than in tertiary hospitals (70% and 40% reimbursement rates, with the same deductibles), but patients still preferred to seek care in tertiary hospitals. Furthermore, the reimbursement rates of health insurance for services received in the JHCs were the same as the services received in the CHCs, which was considered by some administrative staff as a factor to discourage patients to utilize the JHCs. Nevertheless, some chief GPs thought that no extra charges for patients to receive specialists’ services could be an enabling factor to encourage patients to visit JHCs.

### Information environment

Most administrative staff considered that the health information platform was one enabling factor of policy implementation since it effectively connected the health information systems between CHCs and hospitals to better facilitate patient referrals and follow-ups. However, this health information platform was not designed for and tailored to this pilot, and it was still under development with some incompatibilities. Some medical staff said that the connections between health information systems were only available among the health care providers in the same district, and even in the same district, some of their functions were limited with regards to patient information sharing, referral, or follow-up.

*"In our district, we could help our patients to make appointments with the specialists in Hangzhou First People's Hospital on the health information platform, but referring for inpatient services is unavailable. There is another referral platform we could use, through which our health information system connects with four general hospitals. We could directly refer our patients for outpatient or inpatient services there, which is very convenient." (From an administrative staff of CHC).*

### Performance management

Many policymakers reported this pilot was adopted into the annual performance evaluations for the participated CHCs. They also organized specific performance evaluations for the chief GPs every six months. However, some administrative staff in CHCs mentioned that there were few performance evaluations for the participated hospitals as well as the specialists in this pilot, which may discourage their initial participation.

According to our analytic framework, Table 3 summarized the enabling and constraining factors from the design features and supporting environments, which influenced the implementation of the JHCs.

**Table 3:** Enabling and constraining factors in implementation of the Joint Health Centers (JHCs) for chronic care

Aspects	Design and implementation	Enabling factors	Constraining factors
Design features	Governments issued policies specific to integrated care and an explicit implementation plan.	√	
	Governments and other stakeholders designed reasonable key elements of integrated care.	√	
	Policy documents were remained uncertainties in the standards for integration of care and in the criteria for essential medical equipment in the JHCs.		√
Governance	Leadership teams were built from a top-down structure to guarantee efficient governance.	√	
	CHCs can make decisions with regards to care delivery.	√	
Organizational structure and human resources	JHCs were established to link between the primary health care and specialist care, and meanwhile to strengthen the gatekeeper role of the chief GPs.	√	
	Integration mechanism was based on cooperation agreement rather than official contracts between the primary health facilities and the hospitals.		√
	Qualified staffs were selected from the existing pool of health personnel.	√	
	Staffs only worked part-time in this pilot and primarily remained in their original positions.		√
	An innovative capacity-building model, the mentorship system, was developed.	√	
Financing and payment mechanism	Lack of the shared financial incentives and payment mechanism across providers.		√
	Insufficient financial incentives to motivate the medical staffs.		√
	Health insurance system had little influence on patients’ health care seeking behaviors.		√
Information environment	The already-established health information platform was used to connect health information systems between CHCs and municipal hospitals.	√	
	The current system was not designed and tailored specifically to implement this pilot and showed some incompatibilities across systems.		√
Performance management	Performance evaluation was applied to assess performance of CHCs and chief GPs.	√	
	Lack of performance evaluation of hospitals and specialists.		√

Notes: CHCs: community health centers; GPs: general practitioners

### Acceptability on the JHCs’ implementation by the key informants

The administrative staff from providers mentioned that the implementation was well prepared and conducted, and highly in accordance with the implementation plan. Both providers and patients considered this pilot was beneficial to them. The capacity of the medical staff in CHC was thought to be improved, and meanwhile more patients were attracted to accessing CHC after the implementation of JHC.

In patients’ opinion, JHC enabled them to access to the specialists in their communities with improved convenience. They mentioned that they were satisfied with the health services provided in JHC, and they preferred to visit the JHC first when they had chronic disease problems. However, some patients mentioned they still needed to go to tertiary hospitals for prescribing some medicines, which were not in the list of essential medicines of CHCs.

*"We think it is a great project. It is good for our medical staff to improve their capacities, be good for our CHC, and also bring patients for us..." (From an administrative staff of CHC).*

*"I really like the services provided in the JHC. I could access to the specialist here rather than going to the hospital. It is very handy and also not expensive..." (From one diabetes patient).*

*"Before [the implementation of JHC], I only came to CHC for prescribing medicines for my diabetes, however, after that, I started to come to JHC regularly. The chief GP and specialist could provide very professional health services to me. It is very convenient for me now..." (From one diabetes patient).*

*“I like the healthcare provided in JHC, but sometimes, the JHC does not have the medicines the specialist prescribe to me. I have to go the tertiary hospital again for these medicines... (From one patient).*

Nevertheless, several challenges existed during implementation. First of all, it could not guarantee that patients receive frequent specialist services in the JHCs because it was sometimes influenced by the specialists’ workload in the hospitals and the insufficient subsidies for service provision. In some JHCs, it was common for patients to receive outpatient specialist service once per month. In addition, some patients easily missed the sessions or walked in unpunctually for an appointment, as there was no restriction or payment incentive in the health insurance system to influence the behavior of patients. Moreover, mutual referrals between coordinated CHCs and hospitals were somewhat difficult and time-consuming, because beds are not always available in the tertiary hospitals and patients may not be willing to be referred to the coordinated facilities.

*"It is difficult, sometimes to refer the patients to the hospitals because there was no bed available. And it is also challenging to refer patients back to the CHCs since patients refused to be cared in the CHCs..." (From an administrative staff of a hospital).*

### Trends of health care delivery following the JHCs’ implementation

Table 4 presented the change of health care distribution and expenditure before (2011–13) and after (2013–14) the implementation of JHC pilot. The very preliminary results showed that the proportion of outpatient services in CHCs was steadily around 28% during 2011–14, which may be due to that only a small proportion of health care facilities participated in this pilot. The annual change rate of expenditure per outpatient visit in hospitals was steadily around 4–6%, whereas the annual change rate in CHCs during 2013–14 was twice or three times of that during 2011–13. This may indicate more patients with chronic diseases used outpatient services in CHCs. Generally they spent more, and therefore increased expenditures per visit in CHCs.

**Table 4:** Distribution and expenditure of outpatient services at Hangzhou (2011–2014)

	2011	2012	2013	2014
Proportion of outpatient services utilized in CHC (%)	27.13	28.06	28.56	27.57
Expenditure per visit in hospitals (RMB)	225.71	235.10	249.50	262.40
Annual change rate (%)	–	4.16	6.13	5.17
Expenditure per visit in CHC (RMB)	86.02	89.40	94.30	106.00
Annual change rate (%)	–	3.93	5.48	12.41

## Discussion

This was the first study to qualitatively analyze the integrated care pilot in China. Through the qualitative analysis about the perspectives on integrated care pilot from various stakeholders, this study aimed to identify which design features and supporting environments would facilitate or constrain the implementation of integrated care reform in China’s context. Both enabling and constraining factors to the implementation were summarized from the design, supporting environment, and implementation process of the JHCs. Overall, it was apparently the design, governance, and organizational structure and human resources of JHCs that enabled the integrated care reform, indicating that the Hangzhou municipal government put more emphasis on the formation of integrated care. But there still lacked an integrated payment mechanism and a tailored information system to ensure its sustainability. These experiences could be helpful to identify and solve the constraining factors in the future implementation, and provide some insightful evidence for better scale-up of integrated care in China and in other similar contexts.

In the JHCs model, the CHCs were at the leading position and were given sufficient autonomy to decide the integrated care delivery, which enabled CHCs to play a gate-keeper role in the JHCs and significantly motivated their initial participation in the project. In contrast, in the previous pilot models of integrated care in China, hospitals usually led the CHCs and failed to redirect more patients back to CHCs [1]. Therefore, designing the core role of CHCs and giving them sufficient autonomies in the integrated care are vital to the successful development of this project.

The mentorship system between specialists and chief GPs was introduced in the JHC model, which potentially enhanced the capacity and care quality of GPs. The previous studies documented that the poor quality of care in CHCs was the main reason for patients to skip the CHCs to seek care in hospitals in China [1, 45, 46], and well-trained health workers were generally concentrated in hospitals [2, 47, 48]. In the JHC model, this learning-from-practice training model inspired GPs to continuous learning more effectively than the previous training models such as attending workshops or meetings. Meanwhile, a one-on-one relationship between specialists and GPs was built during the mentorship, which further consolidated the cooperation for providing integrated care. Adopting the capacity building for CHCs into the integrated care could be a useful strategy to promote integrated care in the context where the poor capacity of CHCs is a key barrier to deliver health care.

Moreover, the sustainability of choosing medical staff from the existing pool of health personnel in hospitals to work in JHCs would be negatively influenced by their original heavy workloads and inadequate motivations. The previous studies showed that tertiary hospitals were overwhelmed with heavy workloads, and it was very common for one specialist to serve about 100 patients daily in outpatient department [1, 49, 50]. Additionally, there was no sufficient financial incentive to support specialists to work in the JHCs. Therefore, the heavy workloads and lack of additional rewards would negatively affect the specialists’ enthusiasm and serving time devoted in integrated care. Adjusting the workloads of health professionals and sufficient financial incentives are necessary to support them serving in integrated care and its sustainability.

Simultaneously, the JHC model in Hangzhou put a high priority on the healthcare delivery reform, but little changes had been taken place on the health insurance system. Consequently, the health insurance was not a leverage to support the integration of care. For health care providers, there was an absence of shared payment mechanism across providers to encourage them to collaborate, which could hinder the sustainability of this model. For the patients, the current health insurance system had little influence on the health care seeking behaviors, who continuously preferred hospitals to the CHCs and JHCs. Financial incentives should be realigned to take all integrated care providers as a whole, and also guide patients to use CHCs more frequently.

### Limitations

This study had some limitations. First, our study focused on the program implementation using qualitative methods, but did not evaluate the effect of this integrated care model on the performance of the health care delivery system. Future research would collect quantitative data such as electronic medical records to evaluate the effect of this model. Second, the hospitals and CHCs recruited in this study were located in urban areas and were geographically concentrated, which was easy to implement the coordination of care. Thus, the JHC integrated care model may not be generalizable to rural areas or other areas where providers were scattered. Despite these limitations, this study had significant policy implications for China and other countries with poor-quality of primary care to develop an integrated chronic care model.

## Conclusion

As an integrated care pilot, Hangzhou City established the JHCs based on cooperation agreement between CHCs and hospitals to deliver primary and specialty care together for NCD patients. This was designed as a CHC-led delivery system rather than a hospital-oriented system adopted in most previous pilots in China, which enabled CHCs to play as the gate-keeper and care coordinator for the full continuum of services across the health care providers. The Hangzhou municipal government put integrated care a priority, and constructed an efficient design, governance and organizational structure to enable its implementation. An innovative learning-from-practice mentorship system between specialists and GPs was also introduced to improve the capacity of GPs, and it is essential for China and other settings with poor-quality of primary care. However, there remained some constraining factors in the supporting environments that need to be solved in the future. First, the integrated care standards should be clearly stated in the policy design. Second, selecting the existing staff or recruiting new staff for integrated care should be taken into consideration in advance, and sufficient financial incentives should be provided to specialists to serve in integrated care. Third, health insurance should play a proactive role to support the integration of care. Health insurance should adopt the shared payment mechanism across providers to encourage them to collaborate, and also guide patients to use more primary care.

For the application and scaling up of the integrated care reform, the JHC model can be applied in the following context. First, it would be very useful in the settings with poor-quality of primary care. The mentorship between specialists and GPs would improve the quality of primary care. Second, the membership of JHC, such as hospitals and CHCs, should be geographically close to each other. In this situation, specialists from hospitals can easily visit the JHCs to provide the consulting services. Third, health information system is necessary for the JHC, which can achieve patient information sharing, referral, and follow-up within the integrated care network.

## Additional file

Additional file 1: Table S1. An overview of health resources in Hangzhou (2013). **Table S2a.** Characteristics of the sampled key informants in FGDs. **Table S2b.** Characteristics of the sampled key informants in IDIs. **Table S3.** The detailed points for probing in topic guides. (DOCX 28 kb)

## References

[1] Yip W, Hsiao W (2014). Harnessing the privatisation of China’s fragmented health-care delivery. Lancet.

[2] He AJ, Meng Q (2015). An interim interdisciplinary evaluation of China’ s national health care reform: emerging evidence and new perspectives. J. Asian Public Policy.

[3] Qian J (2015). Reallocating authority in the Chinese health system: an institutional perspective. J. Asian Public Policy.

[4] Wu S, Wang C, Zhang G. Has China’s new health care reform improved efficiency at the provincial level? Evidence from a panel data of 31 Chinese provinces. J. Asian Public Policy 2015;8:36–55.

[5] Ministry of Health of China (2011). China health statistical yearbook 2010.

[6] Ministry of Health of China (2012). China health statistical yearbook 2011.

[7] Ministry of Health of China (2013). China health statistical yearbook 2012.

[8] National Health and Family Planning Commission of China (NHFPC) (2014). China health statistical yearbook 2013. Beijing: National Health and family planning Commission of China (NHFPC).

[9] National Health and Family Planning Commission of China (NHFPC) (2015). China health statistical yearbook 2014. Beijing: National Health and family planning Commission of China (NHFPC).

[10] National Health and Family Planning Commission of China (NHFPC) (2016). China health statistical yearbook 2015. Beijing: National Health and family planning Commission of China (NHFPC).

[11] Shi L, Makinen M, Lee D-C, Kidane R, Blanchet N, Liang H (2015). Integrated care delivery and health care seeking by chronically-ill patients - a case-control study of rural Henan province, China. Int J Equity Health.

[12] World Bank. World Bank Open Data [Internet]. Popul. Ages 65 Above. 2016 [cited 2016 Jun 7]. Available from: http://data.worldbank.org/indicator/SP.POP.65UP.TO.ZS

[13] Health and Family Planning Commission of China. The national working plan for NCDs preventation and control (2012–2015) of China [Internet]. 2012 [cited 2016 Jun 7]. Available from: http://www.sda.gov.cn/WS01/CL0852/73135.html

[14] World Health Organisation. Noncommunicable diseases country profiles 2014. Geneva: World Health Organization; 2014.

[15] World Health Organisation. Preventing chronic diseases: a vital investment. Geneva: World Health Organisation; 2005.

[16] Xu J, Pan R, Pong RW, Miao Y, Qian D. Different models of hospital–community health Centre collaboration in selected cities in China: a cross-sectional comparative study. Int J Integr Care. 2016;16:18. 10.5334/ijic.2456 PMC5015528 27616952

[17] Leichsenring K. Developing integrated health and social care services for older persons in Europe. Int J Integr Care. 2004;4:e10. 10.5334/ijic.107 PMC1393267 16773149

[18] Manthorpe J. Steering integrated care in England and the Netherlands: the case of dementia care: a neo-institutionalist comparative study. Int J Integr Care. 2005;5:e26.

[19] Mur-Veeman I, van Raak A, Paulus A. Comparing integrated care policy in Europe: does policy matter? Health Policy Amst Neth 2008;85:172–183. 10.1016/j.healthpol.2007.07.008 17767975

[20] World Health Organisation (2008). Making health system work: intergated health services-what and why?.

[21] Armitage GD, Suter E, Oelke ND, Adair CE (2009). Health systems integration: state of the evidence. Int J Integr Care.

[22] Shaw S, Rosen R (2011). An overview of integrated care in the NHS: what is integrated care?.

[23] Nolte E, Pitchforth E (2014). What is the evidence on the economic impact of integrated care. Denmark: European Observatory on Health Systems and Policies.

[24] Ouwens M, Wollersheim H, Hermens R, Hulscher M, Grol R (2005). Integrated care programmes for chronically ill patients: a review of systematic reviews. Int J Qual Health Care.

[25] Dudley L, Garner P. Strategies for integrating primary health services in low- and middle-income countries at the point of delivery. Cochrane Database Syst Rev. 2011;7:CD003318. 10.1002/14651858.CD003318.pub3 PMC6703668 21735392

[26] Ham CJ, Silva DD (2009). Integrating care and transforming Community Services: what works? where next?. Birmingham: Health Services Management Centre, University of Birmingham.

[27] Øvretveit J, Hansson J, Brommels M (2010). An integrated health and social care organisation in Sweden: creation and structure of a unique local public health and social care system. Health Policy Amst Neth.

[28] Shi M (2013). The progress and challenges of vertical ragional medical consortium in China. Chin J Health Policy.

[29] Zhang H, Du Y, Wang H (2015). Analysis on cases of vertical integration of medical resources. Med Philos.

[30] Liu Q, Dai T, Wang X (2013). Research on patterns and policy of hospital and community health resources interaction and integration in China. China J Hosp Adminitration.

[31] Wang X, Birch S, Zhu W, Ma H, Embrett M, Meng Q (2016). Coordination of care in the Chinese health care systems: a gap analysis of service delivery from a provider perspective. BMC Health Serv Res.

[32] Health and Family Planning Commission of Hangzhou. The 12th Five-Year Plan of Health Development of Hangzhou city [Internet]. 2011 [cited 2016 Jun 9]. Available from: http://www.hzwsjsw.gov.cn/ghjh/16196.jhtml

[33] Health and Family Planning Commission of Hangzhou. The working plan of Deepening Health Reform in Hangzhou 2012 [Internet]. [cited 2016 Jun 9]. Available from: http://www.hangzhou.gov.cn/art/2012/7/5/art_933545_304502.html

[34] Health and Family Planning Commission of Hangzhou. The announcement of promoting the high quality medical resources sharing project between municipal hospitals and community health service centers [Internet]. 2014 [cited 2016 Jun 9]. Available from: http://www.hangzhou.gov.cn/art/2014/1/24/art_807362_1693.html

[35] Health and Family Planning Commission of Hangzhou. The ten health priorities serving for people in Hangzhou 2014 [Internet]. 2014 [cited 2016 Jun 27]. Available from: http://www.hzwsjsw.gov.cn/bmwj/32005.jhtml

[36] He Z, Jin Y, Zhang Z. The role of 3-2-1 health care delivery model in community health. China J Hosp Adminitration. 2008;24

[37] Zhao D. The study of vertical integration of medical resources in shanghai. Shanghai: Fudan Univeristy; 2008.

[38] Lin F, Su F, Wu B, Xu Q (2013). The perceptions on the development of the regional medical consortium in Zhenjiang. Chin J Hosp Adm.

[39] Tang Z, Jin Q, Wang Y, Di X, Zhang C, Zhao Y (2008). The analysis of regional vertical integration of healthcare in shanghai. Chin Hosp Manag.

[40] Zheng Z (2011). The perceptions on the implementation of regional medical consortium in Shanghai.

[41] World Bank. Protocol to guide case study research on people centered health care (PCHC) in China. World Bank; 2014.

[42] Hangzhou Statistical Information Net. Statistical Yearbook of Hangzhou 2014 [Internet]. Stat. Yearb. Hangzhou 2014. 2014 [cited 2016 Jun 9]. Available from: http://www.yearbookchina.com/navibooklist-N2014110006-1.html.

[43] Hangzhou Statistical Information Net. General situation of Hangzhou city [Internet]. Gen. Situat. Hangzhou City. 2015 [cited 2016 Jun 9]. Available from: http://www.hangzhou.gov.cn/art/2016/3/24/art_805865_663727.html.

[44] Health and Family Planning Commission of Zhejiang (2014). Zhejiang health statistical yearbook 2014.

[45] Yip W, Hsiao WC (2015). What drove the cycles of Chinese health system reforms?. Health Syst Reform.

[46] Sun Z, Wang S, Barnes SR. Understanding congestion in China’s medical market: an incentive structure perspective. Health Policy Plan. 2016;31:390-403. 10.1093/heapol/czv062 26185181

[47] Meng Q, Yuan J, Jing L, Zhang J (2009). Mobility of primary health care workers in China. Hum Resour Health.

[48] Eggleston K, Ling L, Qingyue M, Lindelow M, Wagstaff A (2008). Health service delivery in China: a literature review. Health Econ.

[49] Li Q, Xie P (2013). Outpatient workload in China. Lancet.

[50] Yip WC-M, Hsiao W, Meng Q, Chen W, Sun X (2010). Realignment of incentives for health-care providers in China. Lancet Lond Engl.

