# Supplementary material for: Integrated care reform in urban China: a qualitative study on design, supporting environment and implementation
Source: Int J Equity Health. 2017 Oct 25;16:185. doi: 10.1186/s12939-017-0686-8 (PMC5657104; doi:10.1186/s12939-017-0686-8)
Supplement: Additional file 1: Table S1. — An overview of health resources in Hangzhou (2013). Table S2a. Characteristics of the sampled key informants in FGDs. Table S2b. Characteristics of the sampled key informants in IDIs. Table S3. The detailed points for probing in topic guides. (DOCX 28 kb) [file 12939_2017_686_MOESM1_ESM.docx]

**Additional file 1: Table S1. An overview of health resources in Hangzhou (2013)**

| **Indicators** | **2013** |
| --- | --- |
| Total number of health facilities | 4139 |
| Number of hospitals | 208 |
| Number of community health facilities | 1266 |
| Community health centers | 127 |
| Community health stations | 1139 |
| Number of maternal and child health facilities | 9 |
| Number of centers of diseases control | 15 |
| Number of health supervision facilities | 16 |
| Number of health professionals per 1000 people | 11.09 |
| (Assistant) Physicians | 4.20 |
| Nurses | 4.39 |
| Ratio of physicians/nurses | 0.96 |
| Number of medical beds per 1000 people | 7.37 |
| Hospitals | 6.60 |

**Additional file 1: Table S2a. Characteristics of the sampled key informants in FGDs**

| **No.** | **Organization** | **Type of key informants** | **Age** | **Gender** | **Working experiences** |
| --- | --- | --- | --- | --- | --- |
| FGD 1 | Zhejiang Provincial Development and Reform Commission | Policy maker | 49 | Male | 6 years in current position |
|  | Zhejiang Provincial Health and Family Planning Commission | Policy maker | 51 | Male | 5 years in current position |
|  | Zhejiang Provincial Department of Finance | Policy maker | 50 | Female | 5 years in current position |
|  | Zhejiang Provincial Department of Human Resource and Social Security | Policy maker | 48 | Male | 4 years in current position |
| FGD 2 | Hangzhou Development and Reform Commission | Policy maker | 48 | Female | 3 years in current position |
|  | Hangzhou Health and Family Planning Commission | Policy maker | 52 | Male | 7 years in current position |
|  | Hangzhou Health and Family Planning Commission | Policy maker | 45 | Female | 4 years in current position |
|  | Hangzhou Finance Bureau | Policy maker | 50 | Male | 5.5 years in current position |
|  | Hangzhou Finance Bureau | Policy maker | 46 | Male | 4.5 years in current position |
|  | Hangzhou Human Resources and Social Security Bureau | Policy maker | 47 | Male | 5 years in current position |
| FGD 3 | Hangzhou Center for Disease Control and Prevention | Policy maker | 40 | Female | 4.5 years in current position |
|  |  | Policy maker | 41 | Male | 3 years in current position |
|  |  | Policy maker | 40 | Male | 5 years in current position |
| FDG 4 | Tertiary hospital | Administrative Staff | 44 | Female | 4 years in current position |
|  |  | Administrative Staff | 48 | Female | 5 years in current position |
|  |  | Administrative Staff | 48 | Male | 6 years in current position |
|  |  | Administrative Staff | 42 | Male | 4.5 years in current position |
|  |  | Administrative Staff | 45 | Male | 4 years in current position |
|  |  | Administrative Staff | 40 | Male | 3 years in current position |
| FGD 5 | Tertiary hospital | Medical staff | 39 | Female | 7 years in current position |
|  |  | Medical staff | 43 | Female | 9 years in current position |
|  |  | Medical staff | 41 | Male | 8 years in current position |
|  |  | Medical staff | 37 | Female | 6 years in current position |
|  |  | Medical staff | 42 | Female | 7.5 years in current position |
|  |  | Medical staff | 40 | Male | 8 years in current position |
| FGD 6 | Community health center A | Administrative Staff | 50 | Male | 6.5 years in current position |
|  |  | Administrative Staff | 48 | Male | 7 years in current position |
|  |  | Administrative Staff | 49 | Male | 5 years in current position |
| FGD 7 | Community health center A | Medical staff (GP) | 45 | Female | 18 years in current position |
|  |  | Medical staff (GP) | 39 | Female | 10 years in current position |
|  |  | Medical staff (nurse) | 50 | Female | About 20 years in current position |
| FGD 8 | Community health center B | Administrative Staff | 45 | Female | 8 years in current position |
|  |  | Administrative Staff | 42 | Female | 7 years in current position |
|  |  | Administrative Staff | 39 | Male | 5.5 years in current position |
| FGD 9 | Community health center B | Medical staff (GP) | 44 | Male | 12 years in current position |
|  |  | Medical staff (GP) | 37 | Female | 10.5 years in current position |
|  |  | Medical staff (nurse) | 48 | Female | 19 years in current position |

**Additional file 1: Table S2b. Characteristics of the sampled key informants in IDIs**

| **No.** | **Organization** | **Type of key informants** | **Age** | **Gender** | **Working experiences** |
| --- | --- | --- | --- | --- | --- |
| IDI 1 | Tertiary hospital | Administrative Staff (director) | 48 | Male | 4 years in current position |
| IDI 2 | Community health center A | Administrative Staff (director) | 50 | Male | 6 years in current position |
| IDI 3 | Community health center B | Administrative Staff (director) | 52 | Male | 8 years in current position |
| IDI 4 | Community health center A | Medical staff (chief GP) | 47 | Female | 10 years in current position |
| IDI 5 | Community health center B | Medical staff (chief GP) | 49 | Male | 6.5 years in current position |
| IDI 6 | Community health center A | Patient (Hypertension) | 55 | Male | Not applicable |
| IDI 7 | Community health center A | Patient (Hypertension) | 70 | Male | Not applicable |
| IDI 8 | Community health center A | Patient (Diabetes) | 69 | Female | Not applicable |
| IDI 9 | Community health center A | Patient (Diabetes, hypertension) | 68 | Female | Not applicable |
| IDI 10 | Community health center B | Patient (Hypertension) | 70 | Female | Not applicable |
| IDI 11 | Community health center B | Patient (Diabetes) | 65 | Male | Not applicable |
| IDI 12 | Community health center B | Patient (Diabetes) | 72 | Female | Not applicable |
| IDI 13 | Community health center B | Patient (Diabetes, hypertension) | 60 | Female | Not applicable |

**Additional file 1: The detailed points for probing in topic guides**

1. **Design features**
   1. Basic information (title, objectives, launch date, lead organization, location/sites)
   2. Target population, eligibility criteria
   3. Integrated providers (number, structure)
   4. Workflow
   5. Scope of services
   6. Integration mechanism
2. **Supporting environment (policy, finance, and institutional environment)**
   1. Policy framework
   2. Governance

- Existence of supervisory structure in support of integrated care
- Incentives for achieving integration (positive or negative)
- Decision rights among participating providers
  1. Finance and payment mechanisms
- Main payment mechanism
- Additional financing to support implementation
  1. Performance management
- Performance accountability mechanism
  1. Information environment
- Information transfer among providers
  1. Organization structure and human resources
- Degree of support among stakeholder institutions
- Measures taken to achieve support or consensus
- Decision making processes
- Organizational structure changes (administrative integration, budgetary integration)
- Communication structure
- Human resources (needs, gaps, solutions)

1. **Implementation**
   1. Preparation
   2. Process
   3. Challenges
